# Increased mtDNA copy number promotes cancer progression by enhancing mitochondrial oxidative phosphorylation in microsatellite-stable colorectal cancer

**DOI:** 10.1038/s41392-018-0011-z

**Published:** 2018-03-30

**Authors:** Xiacheng Sun, Lei Zhan, Yibing Chen, Gang Wang, Linjie He, Qian Wang, Feng Zhou, Fang Yang, Jin Wu, Yousheng Wu, Jinliang Xing, Xianli He, Qichao Huang

**Affiliations:** 1 State Key Laboratory of Cancer Biology and Experimental Teaching Center of Basic Medicine, Fourth Military Medical University, 710032 Xi’an, China; 2 Department of Gastroenterology, Second Affiliated Hospital of Harbin Medical University, 150086 Harbin, China; 3 Center of Genetic & Prenatal Diagnosis, First Affiliated Hospital, Zhengzhou University, 450052 Zhengzhou, China; 4 Department of General Surgery, Tangdu Hospital, Fourth Military Medical University, 710032 Xi’an, China; 5 Department of General Surgery, Huaihai Hospital, Xuzhou Medical University, Xuzhou, 221004 China

## Abstract

Colorectal cancer is one of the leading causes of cancer death worldwide. According to global genomic status, colorectal cancer can be classified into two main types: microsatellite-stable and microsatellite-instable tumors. Moreover, the two subtypes also exhibit different responses to chemotherapeutic agents through distinctive molecular mechanisms. Recently, mitochondrial DNA depletion has been shown to induce apoptotic resistance in microsatellite-instable colorectal cancer. However, the effects of altered mitochondrial DNA copy number on the progression of microsatellite-stable colorectal cancer, which accounts for the majority of colorectal cancer, remain unclear. In this study, we systematically investigated the functional role of altered mitochondrial DNA copy number in the survival and metastasis of microsatellite-stable colorectal cancer cells. Moreover, the underlying molecular mechanisms were also explored. Our results demonstrated that increased mitochondrial DNA copy number by forced mitochondrial transcription factor A expression significantly facilitated cell proliferation and inhibited apoptosis of microsatellite-stable colorectal cancer cells both in vitro and in vivo. Moreover, we demonstrated that increased mitochondrial DNA copy number enhanced the metastasis of microsatellite-stable colorectal cancer cells. Mechanistically, the survival advantage conferred by increased mitochondrial DNA copy number was caused in large part by elevated mitochondrial oxidative phosphorylation. Furthermore, treatment with oligomycin significantly suppressed the survival and metastasis of microsatellite-stable colorectal cancer cells with increased mitochondrial DNA copy number. Our study provides evidence supporting a possible tumor-promoting role for mitochondrial DNA and uncovers the underlying mechanism, which suggests a potential novel therapeutic target for microsatellite-stable colorectal cancer.

## Bowel cancer: Mitochondrial DNA fuels progression

An increase in mitochondrial DNA (mtDNA) in microsatellite stable colorectal cancer (MSSCRC) cells stimulates cell proliferation and prevents cell death. MtDNA copy number is regulated by mitochondrial transcription factor A and both increases and decreases in mtDNA levels have been associated with different types of cancer. A study led by Qichao Huang and Xianli He at the Fourth Military Medical University, China, investigated the effects of altering mtDNA levels in MSSCRC cells on tumor progression in mice. They found that high levels of mtDNA promoted MSSCRC cell survival and metastasis by stimulating mitochondrial oxidative phosphorylation and energy production. Conversely, mtDNA depletion or treatment with the mitochondrial toxin oligomycin reduced the survival and metastasis of MSSCRC cells. These findings suggest that reducing mtDNA copy number could be a useful therapeutic strategy for MSSCRC.

## Introduction

Colorectal cancer (CRC) is one of the leading causes of cancer death worldwide despite recent advances in surgery, radiotherapy, and chemotherapy.^1^ According to its global genomic status, CRC can be classified into two main types: microsatellite stable (MSS, accounting for 90% of CRC cases) and microsatellite instable (MSI, accounting for 10% of CRC cases) tumors.^2^ MSS tumors are characterized by changes in chromosomal copy number and generally show worse prognoses than MSI tumors. By contrast, tumors with MSI accumulate genetic alterations in both coding and noncoding microsatellite repeats, which are widely distributed throughout the genome.^3^ Moreover, the two subtypes exhibit different responses to chemotherapeutic agents through distinctive molecular mechanisms.^4^ Therefore, it is currently accepted that this classification is key in determining the pathological, clinical, and biological characteristics of colon tumors.

As the major source of metabolites and energy in cells, mitochondria often exhibit varying degrees of dysfunction in cancer. For decades, the Warburg effect has been regarded as a hallmark of cancer cells; this effect consists of continuous prevalence of glycolysis and dysregulation of oxidative metabolism.^5^ Interestingly, unlike other types of cancers, CRC relies on mitochondrial oxidative phosphorylation (OXPHOS) as its major source of energy.^6^ Moreover, the content of mitochondria in human CRC tissues has been found to be higher than the content in normal colon mucosa. However, we still do not know precisely how mitochondria are involved in CRC progression.

Mitochondria contain their own genome, which encodes 13 polypeptides involved in the electron transport chain (ETC) and ATP synthase.^7,8^ Cumulative evidence has indicated that variation of mitochondrial DNA (mtDNA) copy number is closely associated with types of cancers. For example, mtDNA is decreased in gastric cancer, breast cancer, hepatocellular carcinoma, non-small cell lung cancer (NSCLC), and renal cell carcinoma.^9–13^ By contrast, mtDNA copy number is increased in other types of cancer, including CRC.^14–18^ Recently, Guo et al. have reported that mtDNA depletion induced by mitochondrial transcription factor A (TFAM) mutation plays a promoting role in tumorigenesis and cisplatin resistance in MSI CRC.^19^ However, the effects of altering mtDNA copy number on the tumor progression of MSS CRC, the majority of CRC, are largely unknown. In the present study, we systematically investigated the functional roles of altered mtDNA copy number in MSS CRC progression and the underlying mechanisms. Our findings demonstrate that increased mtDNA plays a critical role in regulating MSS CRC cell survival and metastasis by promoting mitochondrial OXPHOS, which provides novel evidence for this process as a drug target in MSS CRC treatment.

## Materials and methods

### Cell culture

The human MSS CRC cell lines SW480 and Caco-2^20^ were purchased from ATCC and routinely cultured. SW480 ρ^0^ cells were cultured in the presence of 200 ng/ml ethidium bromide for >20 generations. After 20 generations, mtDNA depletion was confirmed by quantitative reverse transcriptase PCR analysis. The SW480 ρ^0^ cells were maintained in RPMI-1640 supplemented with 10% fetal bovine serum (FBS), 50 µg/ml uridine, and 100 µg/ml sodium pyruvate (ρ^0^ culture medium).

### Knockdown and forced expression of target genes

For knockdown, the specific short hairpin RNA (shRNA) sequence targeting the human TFAM mRNA sequence or a control shRNA was cloned into the pSilencer™ 3.1-H1 puro vector (Ambion, Waltham, MA). For overexpression, the coding sequence of TFAM was amplified from cDNA derived from SW480 cells using the primers listed in Supplementary Table 1 and cloned into the pcDNA™3.1(+) vector (Invitrogen, Waltham, MA). Then the vectors were transfected into CRC cells using the Lipofectamine 2000 reagent (Invitrogen, Waltham, MA) according to the manufacturer’s instructions.

### Detection of mtDNA content by real-time quantitative PCR

Genomic DNA was extracted from CRC cells using the E.Z.N.A. Tissue DNA Kit (Omega BioTek, Norcross, GA). Relative mtDNA copy number was measured by a quantitative real-time PCR-based method as previously described.^21^ Each reaction was optimized and confirmed to be linear within an appropriate concentration range using genomic DNA from a normal sample control.

### Western blot and immunohistochemical (IHC) staining

Western blot and IHC staining were performed as previously described.^22^ A Protein Quantitation Kit (W-6006) was purchased from US Everbright Inc. (Suzhou, China). Primary antibodies against TFAM (1/1000, Abcam), β-actin (1/3000, Beijing TDY BIOTEC), cyt c (1/750, Proteintech), COX4 (1/750, ABGENT), CASP9 (cleaved) (1/1000, Proteintech), and CASP3 (cleaved) (1/750, Proteintech) were used in western blot analysis. A primary antibody against Ki-67 (1:100, Abcam) was used for IHC staining.

### Nude mice xenograft model

BALB/c nude mice (18–22 g) were randomly divided into groups. Xenografts were initiated by subcutaneous injection of 10^7^ CRC cells into the backs of nude mice (*n* = 6). Tumor size, including length (*L*) and width (*W*), was measured using Vernier calipers every 4 days starting on day 7 after transplantation. The tumor volumes were calculated according to the formula (*L*×*W*^2^)/2 and presented as the mean ± SEM. Four weeks later, the mice were sacrificed and tumor nodules were harvested and photographed. For the in vivo tumor-metastasis assay, 5 × 10^5^ CRC cells were injected into the tail veins of BALB/c nude mice (*n* = 6). Forty days after injection, the mice were sacrificed. The study was approved by the animal research ethics committee of the Fourth Military Medical University.

### Determination of in vitro cell proliferation

CRC cells were seeded into 24-well plates at a density of 0.2 × 10^4^ cells/well. The cells were trypsinized and counted at 0, 1, 2, 3, and 4 days. The number of cells was counted using a Countess Automated Cell Counter (Invitrogen, Carlsbad, CA, USA). Each assay was performed in three separate experiments.

### Clone-formation assay

Log-phase SW480 cells or Caco-2 cells were plated in 6-well plates at a density of 10^3^ cells/well or 0.6 × 10^3^ cells/well, respectively. The colonies were stained with Giemsa after 15 days of culture, and then the total number of colonies was counted. Each assay was performed in three separate experiments.

### Ethynyl deoxyuridine (EdU) incorporation assay

The proliferation of cells was analyzed using an EdU Incorporation Assay Kit (Ribobio, Guangdong, China) according to the manufacturer’s instructions. The staining cells were visualized under a fluorescence microscope (Olympus FV1000).

### Terminal deoxynucleotidyl transferase dUTP nick end labeling (TUNEL) assay

For apoptosis analysis in xenograft tissues, a TUNEL assay (Roche, Switzerland) was performed according to the manufacturer’s protocols. Images of TUNEL/DAPI (4,6-diamidino-2-phenylindole)-stained sections were obtained through a fluorescence microscope (DM5000B; Leica, Heerbrugg, Switzerland). The apoptosis index was calculated as the percentage of TUNEL-positive nuclei after at least 500 cells were counted.

### Transwell invasion analysis

For invasion assays, chamber inserts were coated with 200 mg/ml Matrigel and dried overnight under sterile conditions. A cell suspension was placed into the upper chamber. Medium supplemented with 15% FBS was placed in the lower chamber as a chemoattractant. After incubation for 48 h, cells on the lower surfaces of the chambers were fixed and stained with crystal violet, then visualized, and counted.

### Detection of mitochondrial membrane potential, mitochondrial ATP level, and oxygen consumption rate (OCR)

JC-1 dye was purchased from Molecular Probes™ (Invitrogen, USA). Cells were adjusted to a density of 1 × 10^6^/ml and stained with 10 μg/ml JC-1 for 10 min at 37 °C. Cells were then resuspended and analyzed by flow cytometry (Beckman, Fullerton, CA). The Cell Titer-Glo Luminescent Cell Viability Assay kit (Promega, Wisconsin, USA) was used for the ATP assay according to the manufacturer’s instructions. The OCR of CRC cells was evaluated using an oxygen electrode system as previously described.

### Statistical analysis

Experiments were repeated three times where appropriate. Data represents mean ± SEM. SPSS 17.0 software (SPSS, Chicago, IL) was used for all statistical analyses, and *P* < 0.05 was considered significant. Unpaired *t*-tests (two tailed) were used for comparisons between two groups after checking for normal distribution and equal variance of the data.

## Results

### Increased mtDNA copy number promoted the survival of MSS CRC cells both in vitro and in vivo

**Fig. 1:**
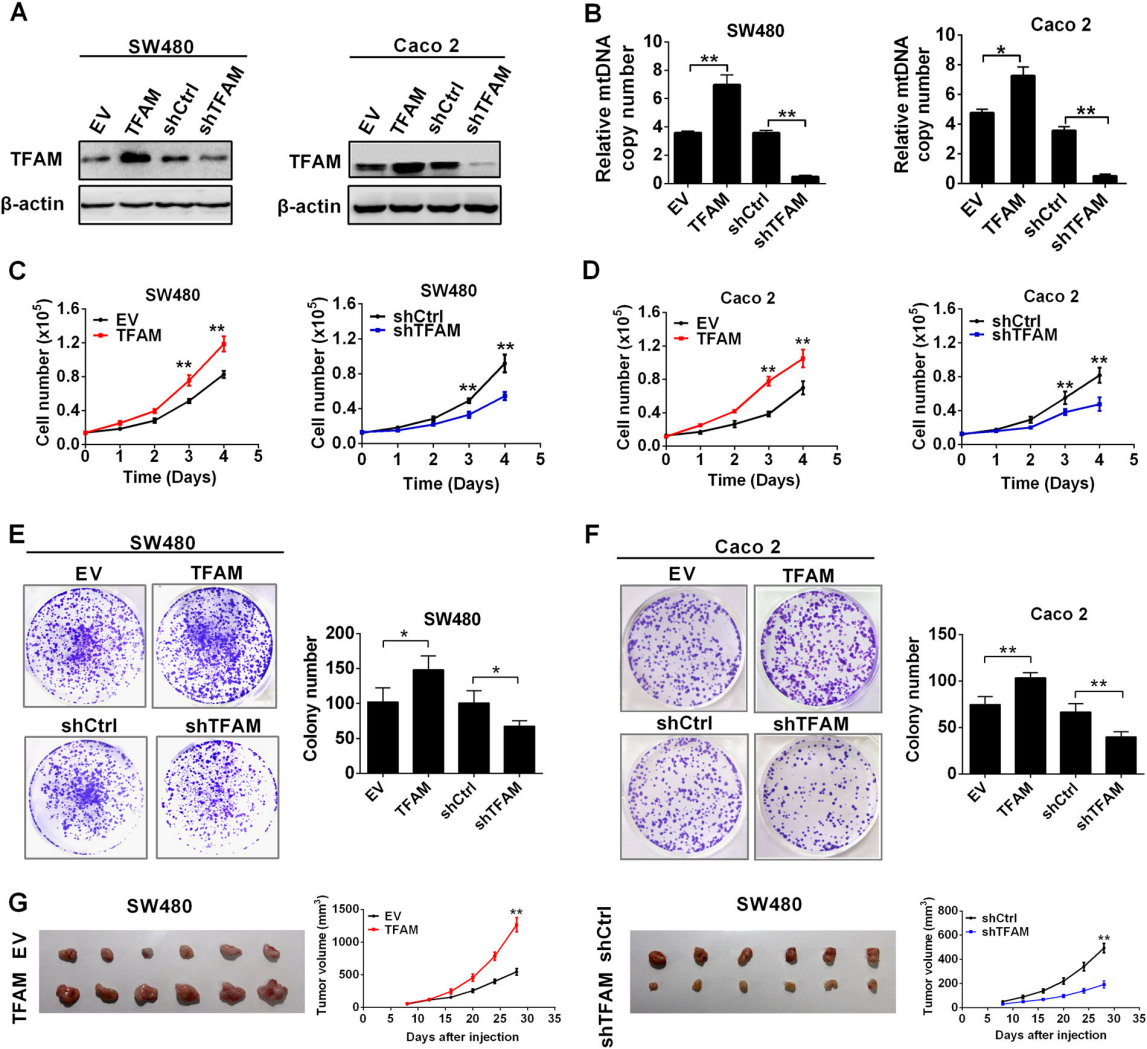
Increased mtDNA copy number promoted the survival of MSS CRC cells. **a** Western blot analyses for TFAM expression in SW480 and Caco-2 cells transfected with shRNA or expression vector. **b** qRT–PCR analyses for mtDNA copy number was performed in SW480 and Caco-2 cells with different TFAM expression levels. The data shown are the mean ± SEM from three separate experiments. **c**, **d** Cell counting of SW480 and Caco-2 cells with TFAM overexpression or knockdown. **e**, **f** Colony-formation assay in SW480 and Caco-2 cells with different treatments as indicated. The data shown are the mean ± SEM from three separate experiments. **g** Tumor growth curves of subcutaneous xenograft tumor models developed from CRC cells with different treatments as indicated (right panel). Tumors from sacrificed mice are also shown in the left panel. TFAM expression vector encoding TFAM, EV empty vector, shTFAM shRNA vector against TFAM, shCtrl control shRNA vector, WT wild type, rho 0 mtDNA deletion. *P < 0.05; **P < 0.01

TFAM is an important regulator of mtDNA replication. Therefore, TFAM was stably overexpressed in MSS CRC cell lines SW480 and Caco-2 to elevate mtDNA copy number, while TFAM stable silencing by shRNA was used to decrease mtDNA content (Fig. 1a, b). Meanwhile, TFAM overexpression promoted cell proliferation and colony formation in both MSS CRC cell lines, whereas TFAM silencing inhibited cell proliferation and colony formation (Fig. 1c–f). We next examined the effects of altered mtDNA copy number on tumor growth in a nude mouse xenograft model. As shown in Fig. 1g, SW480 cells with stable TFAM overexpression exhibited a significantly increased tumorigenic capacity (*P* = 0.004), whereas those with stable TFAM knockdown exhibited a decreased tumorigenic capacity.

### Increased mtDNA copy number promoted cell survival by accelerating cell proliferation and inhibiting apoptosis of HCC cells

**Fig. 2:**
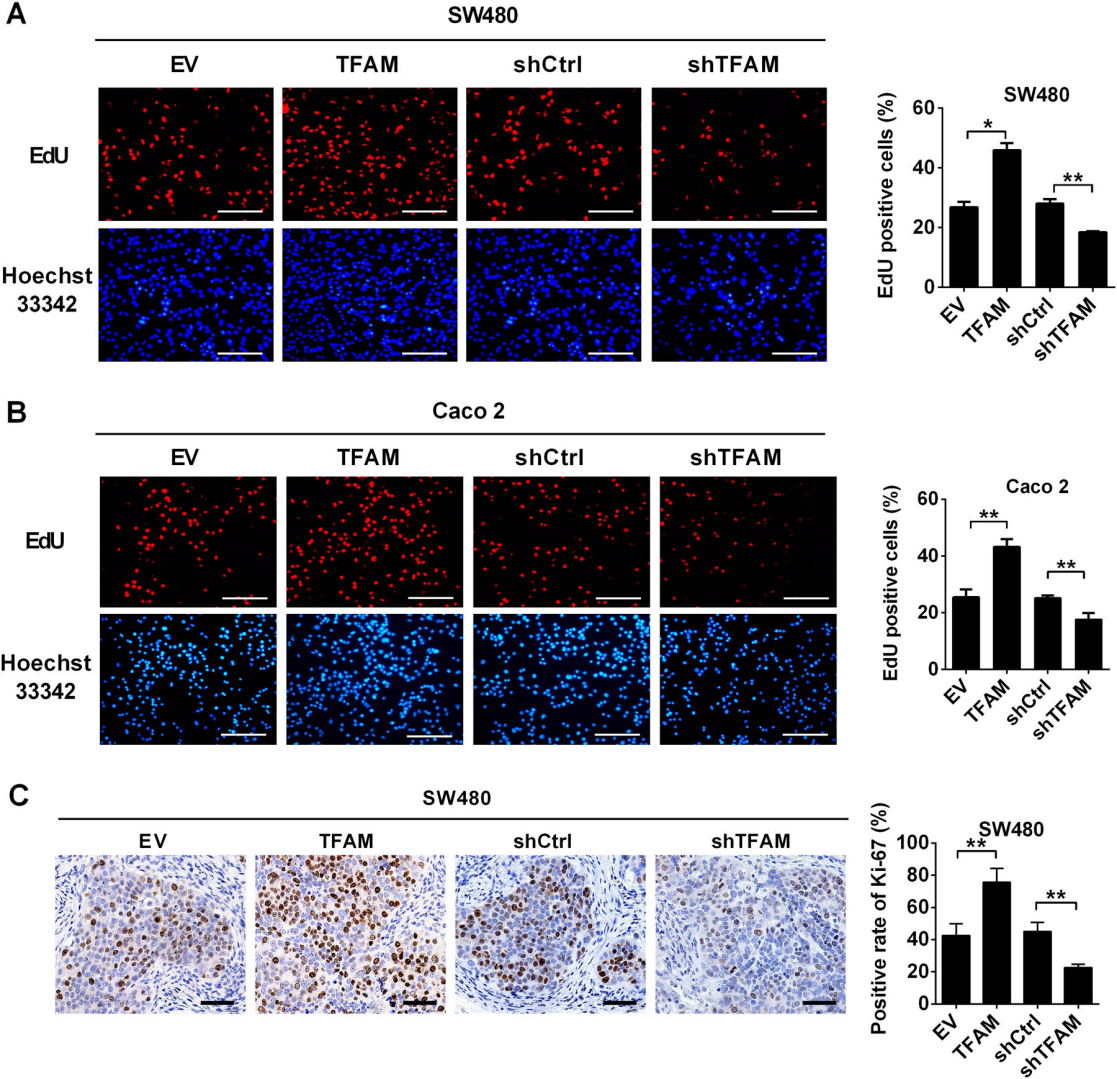
Increased mtDNA copy number promoted cell proliferation of MSS CRC cells. **a**, **b** Cell proliferation was evaluated with an EdU incorporation assay in SW480 and Caco-2 cells with the indicated treatments. Scale bar: 50 μm. **c** Representative immunohistochemical (IHC) staining images of Ki-67 in xenograft tumors developed from SW480 cells that were stably transfected with different expression vectors as indicated. Scale bar: 100 μm. The data shown are the mean ± SEM from three separate experiments. *P < 0.05; **P < 0.01

We further assessed the functional role of altered mtDNA copy number in proliferation and apoptosis of MSS CRC cells. The EdU incorporation assay revealed that MSS CRC cells with TFAM overexpression had higher EdU incorporation rate than control cells. By contrast, MSS CRC cells with TFAM knockdown had lower EdU incorporation rate (Fig. 2a, b). We next investigated the effect of altered mtDNA copy number on tumor cell proliferation in vivo. IHC staining analysis demonstrated that the fraction of Ki-67 (a nuclear proliferation antigen) positive cells was significantly increased in xenograft tumors developed from SW480 cells with TFAM overexpression compared with those in control xenograft tumors (*P* = 0.006). By contrast, TFAM knockdown significantly decreased the intensity of Ki-67 staining in xenograft tumors (*P* = 0.007) (Fig. 2c).

**Fig. 3:**
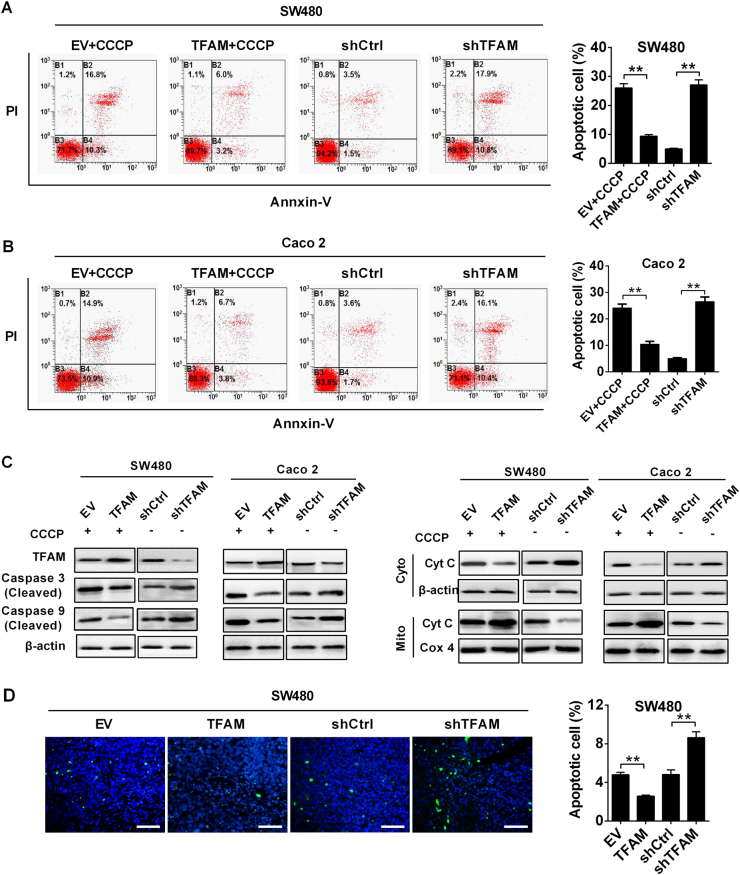
Increased mtDNA copy number inhibited apoptosis of MSS CRC cells. **a**, **b** Apoptosis analysis by flow cytometry in both SW480 and Caco-2 cells with treatment as indicated. Cells stably transfected with EV or TFAM vector were also treated with CCCP (150 μM) for 4 h before apoptosis analysis. **c** Western blot analyses for protein levels of caspase 3/9 and cytochrome C (Cyt C) in CRC cells with the indicated treatments. Cells stably transfected with EV or TFAM vector were also treated with CCCP (150 μM) for 4 h before analysis. β-Actin and Cox4 were used as loading controls for cytoplasm and mitochondria, respectively. Cyto cytoplasm, Mito mitochondria. **d** TUNEL staining in tumor tissues of nude mouse xenograft models developed from different CRC cells stably transfected with different expression vector. Blue: Hoechst 33342; Green: TUNEL-positive nucleus. The data shown are the mean ± SEM from three separate experiments. **P < 0.01

Flow cytometric analysis revealed that increasing the mtDNA copy number by TFAM overexpression remarkably inhibited the apoptosis induced by carbonyl cyanide m-chlorophenyl hydrazone (CCCP, an uncoupler of OXPHOS) in both MSS CRC cell lines, whereas the percentages of total apoptotic cells were significantly higher in cells with TFAM knockdown (Fig. 3a, b). Moreover, cytochrome c release and the cleavage of caspase-9 and caspase-3 were remarkably inhibited by TFAM overexpression upon CCCP treatment but increased by TFAM knockdown (Fig. 3c). Importantly, the forced expression of TFAM significantly reduced TUNEL-positive staining in xenografts (*P* = 0.008), while stable TFAM knockdown increased TUNEL-positive staining (*P* = 0.009) (Fig. 3d).

### Increased mtDNA copy number enhanced the metastatic ability of MSS CRC cells

**Fig. 4:**
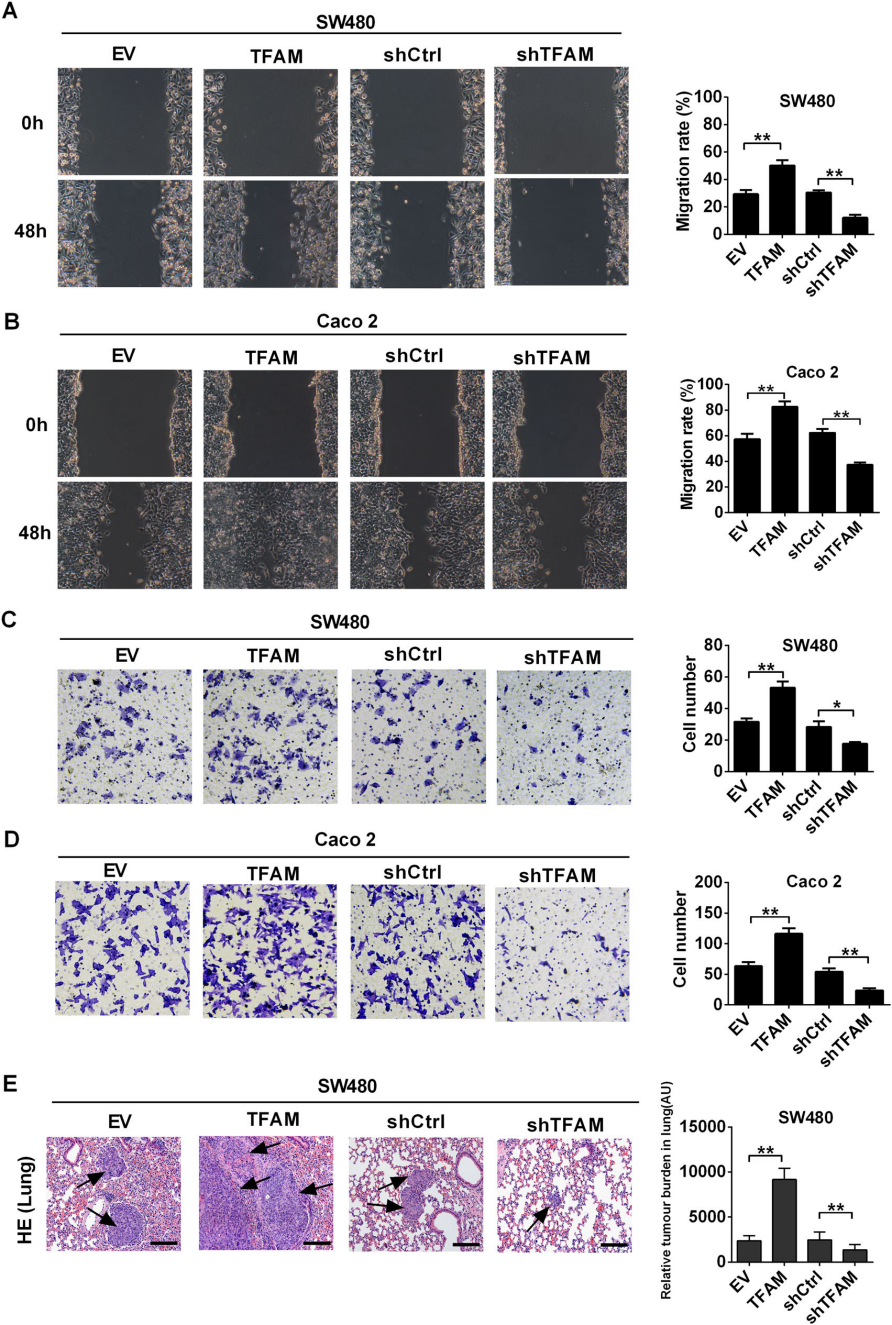
Increased mtDNA copy number promoted metastasis of MSS CRC cells. **a**, **b** Representative images of the wound-healing assay in SW480 and Caco-2 cells with treatment as indicated. **c**, **d** Transwell invasion analysis for CRC cells with treatment as indicated. **e** H&E staining of the lungs from tail-vein-injected mice (left). Quantification of lung metastases (right). The data shown are the mean ± SEM from three separate experiments. *P < 0.05; **P < 0.01

Next, wound healing and transwell invasion assays were performed to assess the effect of increased mtDNA copy number on the migration ability of MSS CRC cells. Our data demonstrated that increased mtDNA copy number mediated by TFAM overexpression promoted the migration and invasion of MSS CRC cells, while decreased mtDNA copy number mediated by TFAM knockdown exhibited the opposite effects (Fig. 4a–d). To further examine the effects of mtDNA copy number on metastasis in vivo, we injected MSS CRC cells into the tail vein of BALB/c nude mice. Our results indicated that the number of micrometastases was much greater in the lungs of mice injected with CRC cells with TFAM overexpression (*P* = 0.008). By contrast, TFAM knockdown significantly reduced the number of micrometastases in the lungs (*P* = 0.006) (Fig. 4e). Taken together, our data demonstrate that the increased mtDNA copy number promotes the metastasis of MSS CRC cells.

### Depletion of mtDNA prevented the survival and metastasis of MSS CRC cells

**Fig. 5:**
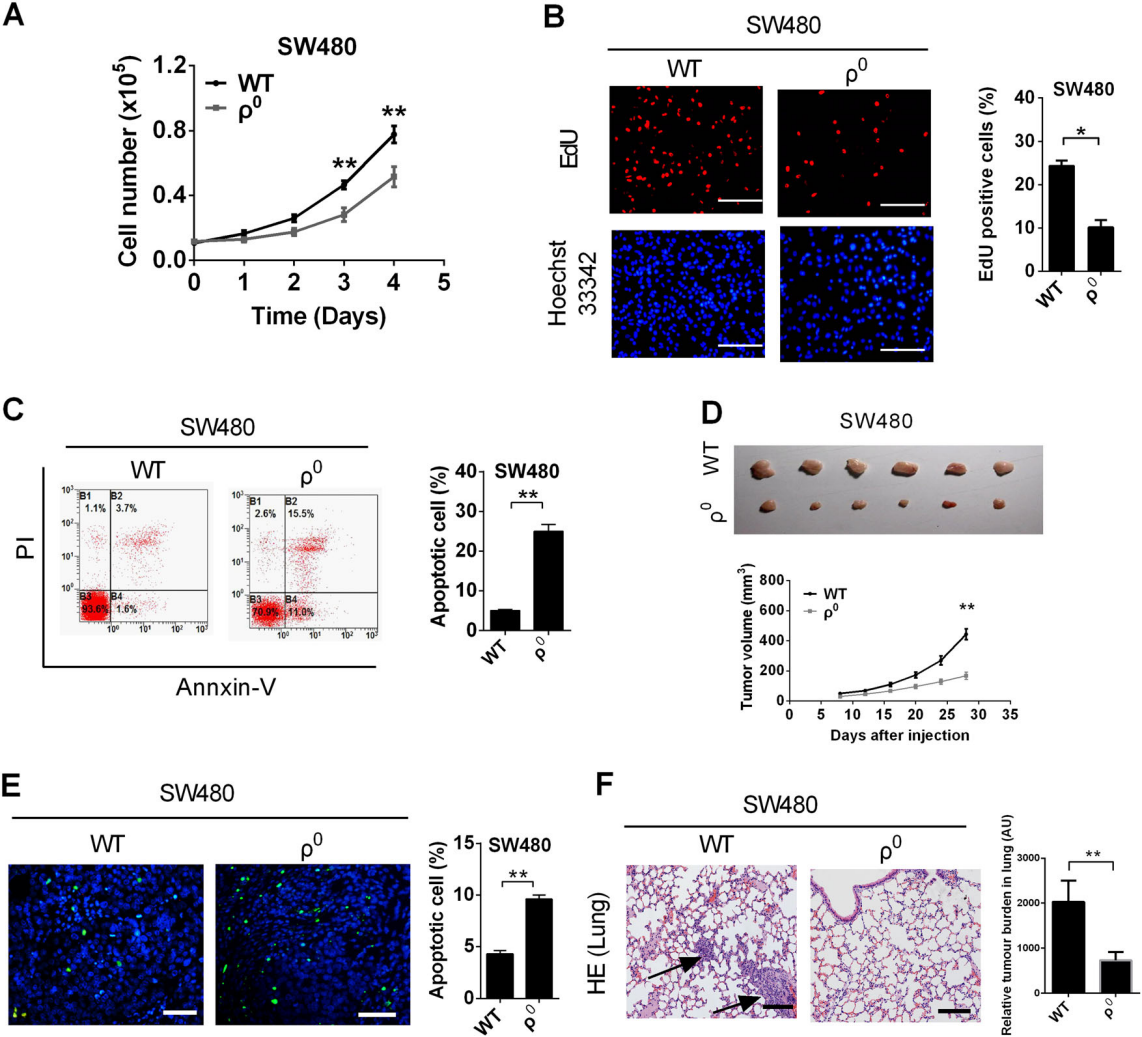
mtDNA depletion prevented the survival and metastasis of MSS CRC cells. **a** Cell counting of SW480 cell with TFAM treatment as indicated. **b** Cell proliferation was evaluated by an EdU incorporation assay in SW480 cells with the indicated treatments. Scale bar: 50 μm. **c** Apoptosis analysis by flow cytometry in SW480 cells with treatment as indicated. Cells were treated with CCCP (150 μM) for 4 h before apoptosis analysis. **d** Tumor growth curves of subcutaneous xenograft tumor models developed from SW480 cells with different treatments as indicated (lower panel). Tumors from sacrificed mice were dissected 28 days after transplantation and are also shown in the upper panel. **e** TUNEL staining in tumor tissues of nude mouse xenograft models developed from SW480 cells with different treatments as indicated. Blue: Hoechst 33342; Green: TUNEL-positive nucleus. **f** H&E staining of the lungs from tail-vein-injected mice (left). Quantification of lung metastases (right). The data shown are the mean ± SEM from three separate experiments. *P < 0.05; **P < 0.01

To further clarify the role of altered mtDNA copy number in the survival and metastasis of MSS CRC cells, we established an SW480 cell line with mtDNA depletion (ρ^0^ cell) (Fig. S1A). Our results demonstrated that the proliferation was significantly impaired in ρ^0^ cells compared to control cells (*P* = 0.007) (Fig. 5a). Additionally, the EdU incorporation rate and colony-formation ability were decreased in ρ^0^cells (Fig. 5b and S1B), whereas the percentages of total apoptotic cells (Fig. 5c), cytochrome c release, cleaved caspase-9, and caspase-3 were higher in ρ^0^ cells (Fig. S1C). Moreover, metastatic ability was also impaired in ρ^0^ cells (Fig. S1D and S1E). Xenograft tumors developed from ρ^0^ cells exhibited significantly increased TUNEL-positive staining (*P* = 0.009) (Fig. 5d) but decreased tumorigenesis capacity and a smaller fraction of Ki-67-positive cells (Fig. 5e and Fig. S1F) than the tumors developed from control cells. Consistently, the number of micrometastases was much lower in the lungs of mice injected with ρ^0^ cells (Fig. 5f).

### Increased mtDNA copy number promoted cell survival and metastasis by enhancing mitochondrial OXPHOS function in MSS CRC

**Fig. 6:**
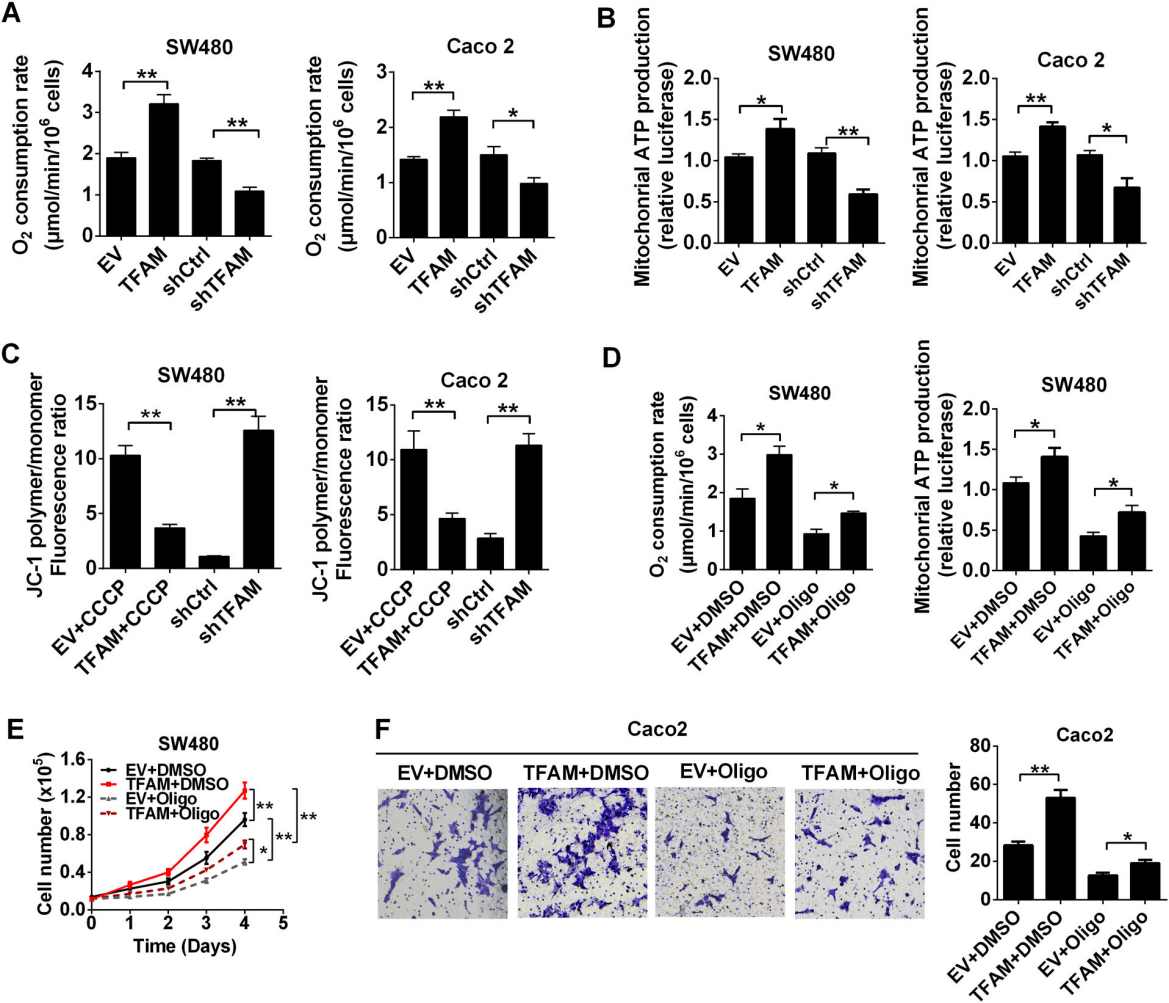
Increased mtDNA copy number promoted cell survival and metastasis by promoting mitochondrial OXPHOS function in MSS CRC. **a** Oxygen consumption rates (OCR) were measured with a liquid-phase oxygen electrode in SW480 and Caco-2 cells with the indicated treatments. **b** Mitochondrial ATP levels were measured in treated SW480 and Caco-2 cells. **c** Depolarization of mitochondrial membrane potential was analyzed by JC-1 staining in treated CRC cells. Cells stably transfected with EV or TFAM vector were also treated with CCCP (150 μM) for 4 h before mitochondrial membrane potential analysis. **d** OCR and mitochondrial ATP level were measured in SW480 cells transfected with EV or TFAM vector. Cells were treated with Oligo (Oligomycin) for 12 h before analysis. **e**, **f** Cell proliferation and invasion were evaluated by numerical counts of SW480 cells with different treatments as indicated. The data shown are the mean ± SEM from three separate experiments. *P < 0.05; **P < 0.01

To explore the underlying mechanism whereby increased mtDNA copy number promotes MSS CRC cell survival and metastasis, we further examined whether mtDNA copy number causes alterations in mitochondrial function. Our results showed that OCR and mitochondrial ATP production were significantly promoted by TFAM overexpression, while TFAM knockdown exhibited the opposite effects (Fig. 6a, b). Furthermore, flow cytometric analysis indicated that TFAM overexpression preserved the mitochondrial membrane potential of MSS CRC cells treated with CCCP, whereas TFAM knockdown exhibited the opposite effects (Fig. 6c and Fig. S2). Notably, these effects could be inhibited by oligomycin, an inhibitor of ATP synthase. More importantly, the enhanced survival and metastasis mediated by TFAM overexpression were notably repressed by oligomycin treatment (Fig. 6e, f). Taken together, these data suggest that the increased mtDNA copy number may promote cell survival and metastasis, partially by enhancing mitochondrial OXPHOS function in MSS CRC cells.

## Discussion

Most free energy is produced by mitochondrial OXPHOS, during which electrons derived from NADH and FADH2 are transported to the ETC to generate ATP. In human cells, mtDNA includes 13 genes, which encode ETC components and play key roles in supporting ETC activity.^23^ Interestingly, alteration of mtDNA copy number has been observed in many types of cancers, including CRC.^24^ Moreover, accumulating evidence has implied that mtDNA copy number alterations play a crucial role in the development of CRC. Shi et al. have reported that mtDNA copy number is significantly increased CRC tissues. Moreover, this increase is particularly marked in stages I and II, indicating that mtDNA copy number plays an important role during the initiation of CRC.^25^ Wen et al. have demonstrated that increased mtDNA copy number mediated by p53-upregulated TFAM is significantly related to advanced Tumor, Node, Metastasis stages, positive lymph nodes, and low 5-year survival rate in patients with CRC.^26^ Therefore, it seems likely that increased mtDNA copy number would promote the progression of CRC. In fact, in the present study, we found that increased mtDNA copy number significantly promoted MSS CRC cell survival by promoting cell proliferation and inhibiting apoptosis. In agreement with our findings, a series of previous studies have reported that increased mtDNA copy number promotes the survival and apoptosis resistance of cancer cells. For example, Hayashi et al. have shown that the growth of HeLa cells with mtDNA depletion is impeded and can be restored by reintroduction of mtDNA from normal human fibroblasts.^27^ Moreover, Mizumachi et al. have demonstrated that increased mtDNA induces acquired docetaxel resistance in head and neck cancer cells.^28^ In addition, our study found that increased mtDNA copy number promoted cell metastasis in vitro and in vivo in MSS CRC. Consistently, Xu et al. have shown that mtDNA copy number is increased in NSCLC cells following the induction of epithelial–mesenchymal transition by transforming growth factor-β1.^29^

Compared to MSS tumors, CRC with MSI represents a distinct molecular pathway for colorectal carcinogenesis because of the deficiency in DNA mismatch repair.^30^ In this regard, CRC cells with different microsatellite status may harbor different mtDNA content patterns and different subsequent metabolic patterns. Indeed, several previous studies have demonstrated that mtDNA copy number is much lower in MSI CRC tissues (or cell lines) than in MSS CRC tissues (or cell lines).^19,31^ Moreover, MSI CRC samples have higher levels of lactate and lower levels of glucose than MSS samples, suggesting that MSI cancer cells rely on glycolysis, whereas MSS cells favor OXPHOS to support their malignancy. Consistently, mtDNA reduction in MSI CRC cells was shown to promote cell proliferation and chemoresistance.^19^ By contrast, our data demonstrated that increased mtDNA copy number in MSS CRC significantly promoted tumor progression by upregulating OXPHOS function. Therefore, we suspect that the adaptive needs of opposite metabolism patterns may contribute to the opposite roles of mtDNA content in the survival of MSI and MSS CRC cells. However, future studies are warranted to explore its underlying molecular mechanisms.

In summary, our findings demonstrate that increased mtDNA copy number significantly promotes cell proliferation, apoptosis resistance, and metastasis of MSS CRC by upregulating mitochondrial OXPHOS, which provides novel evidence for this process as a drug target in MSS CRC treatment.

## Electronic supplementary material

Supplementary information(DOCX 1052 kb)

## References

[1] Ogino S, Goel A (2008). Molecular classification and correlates in colorectal cancer. J. Mol. Diagn..

[2] Jass JR (2007). Classification of colorectal cancer based on correlation of clinical, morphological and molecular features. Histopathology.

[3] Lengauer C, Kinzler KW, Vogelstein B (1997). DNA methylation and genetic instability in colorectal cancer cells. Proc. Natl. Acad. Sci. USA.

[4] Dienstmann R (2017). Consensus molecular subtypes and the evolution of precision medicine in colorectal cancer. Nat. Rev. Cancer.

[5] Lu J, Tan M, Cai Q (2015). The Warburg effect in tumor progression: mitochondrial oxidative metabolism as an anti-metastasis mechanism. Cancer Lett..

[6] Zheng J (2012). Energy metabolism of cancer: glycolysis versus oxidative phosphorylation (Review). Oncol. Lett..

[7] Attardi G, Schatz G (1988). Biogenesis of mitochondria. Annu. Rev. Cell. Biol..

[8] Pfeiffer T, Schuster S, Bonhoeffer S (2001). Cooperation and competition in the evolution of ATP-producing pathways. Science.

[9] Wu CW (2005). Mitochondrial DNA mutations and mitochondrial DNA depletion in gastric cancer. Genes. Chromosomes Cancer.

[10] Mambo E (2005). Tumor-specific changes in mtDNA content in human cancer. Int. J. Cancer.

[11] Yin PH (2004). Alteration of the copy number and deletion of mitochondrial DNA in human hepatocellular carcinoma. Br. J. Cancer.

[12] Lin CS, Wang LS, Tsai CM, Wei YH (2008). Low copy number and low oxidative damage of mitochondrial DNA are associated with tumor progression in lung cancer tissues after neoadjuvant chemotherapy. Interact. Cardiovasc. Thorac. Surg..

[13] Meierhofer D (2004). Decrease of mitochondrial DNA content and energy metabolism in renal cell carcinoma. Carcinogenesis.

[14] Egan K, Kusao I, Troelstrup D, Agsalda M, Shiramizu B (2010). Mitochondrial DNA in residual leukemia cells in cerebrospinal fluid in children with acute lymphoblastic leukemia. J. Clin. Med. Res..

[15] Lin CS (2010). The role of mitochondrial DNA alterations in esophageal squamous cell carcinomas. J. Thorac. Cardiovasc. Surg..

[16] Kim MM (2004). Mitochondrial DNA quantity increases with histopathologic grade in premalignant and malignant head and neck lesions. Clin. Cancer Res..

[17] Wang Y, Liu VW, Xue WC, Cheung AN, Ngan HY (2006). Association of decreased mitochondrial DNA content with ovarian cancer progression. Br. J. Cancer.

[18] Mizumachi T (2008). Increased distributional variance of mitochondrial DNA content associated with prostate cancer cells as compared with normal prostate cells. Prostate.

[19] Guo J (2011). Frequent truncating mutation of TFAM induces mitochondrial DNA depletion and apoptotic resistance in microsatellite-unstable colorectal cancer. Cancer Res..

[20] Ahmed D (2013). Epigenetic and genetic features of 24 colon cancer cell lines. Oncogenesis.

[21] Xing J (2008). Mitochondrial DNA content: its genetic heritability and association with renal cell carcinoma. J. Natl. Cancer Inst..

[22] Huang Q (2016). Increased mitochondrial fission promotes autophagy and hepatocellular carcinoma cell survival through the ROS-modulated coordinated regulation of the NFKB and TP53 pathways. Autophagy.

[23] Huang Q (2014). CD147 promotes reprogramming of glucose metabolism and cell proliferation in HCC cells by inhibiting the p53-dependent signaling pathway. J. Hepatol..

[24] Yu M (2011). Generation, function and diagnostic value of mitochondrial DNA copy number alterations in human cancers. Life Sci..

[25] Feng S, Xiong L, Ji Z, Cheng W, Yang H (2011). Correlation between increased copy number of mitochondrial DNA and clinicopathological stage in colorectal cancer. Oncol. Lett..

[26] Wen S (2016). p53 increase mitochondrial copy number via up-regulation of mitochondrial transcription factor A in colorectal cancer. Oncotarget.

[27] Hayashi J, Takemitsu M, Nonaka I (1992). Recovery of the missing tumorigenicity in mitochondrial DNA-less HeLa cells by introduction of mitochondrial DNA from normal human cells. Somat. Cell Mol. Genet..

[28] Mizumachi T (2008). Increased mitochondrial DNA induces acquired docetaxel resistance in head and neck cancer cells. Oncogene.

[29] Xu Y, Lu S (2015). Transforming growth factor-beta1-induced epithelial to mesenchymal transition increases mitochondrial content in the A549 non-small cell lung cancer cell line. Mol. Med. Rep..

[30] Boland CR, Goel A (2010). Microsatellite instability in colorectal cancer. Gastroenterology.

[31] Lièvre A (2006). Mitochondrial DNA copy number in colorectal cancer cells is dependant of nuclear microsatellite instability status. Cancer Res..

