# Supplementary material for: Increased mtDNA copy number promotes cancer progression by enhancing mitochondrial oxidative phosphorylation in microsatellite-stable colorectal cancer
Source: Signal Transduct Target Ther. 2018 Mar 30;3:8. doi: 10.1038/s41392-018-0011-z (PMC5878831; doi:10.1038/s41392-018-0011-z)
Supplement: Supplementary file 1 — Supplementary information(DOCX 1052 kb) [file 41392_2018_11_MOESM1_ESM.docx]

**Increased mtDNA copy number promotes mitochondrial oxidative phosphorylation (OXPHOS) and MSS colorectal cancer progression**

Xiacheng Sun^1#^, Lei Zhan^2#^, Yibing Chen^3^, Gang Wang^4^, Linjie He^1^,Qian Wang^4^,

Zhou Feng^5^, Fang Yang^1^,Jin Wu^1^,Yousheng Wu^1^,Jinliang Xing^1^,Xianli He^4^*, Qichao Huang^1^*

^1^State Key Laboratory of Cancer Biology and Experimental Teaching Center of Basic Medicine, Fourth Military Medical University, Xi’an, 710032, China

^2^Department of Gastroenterology, Second Affiliated Hospital of Harbin Medical University, Harbin, 150086, China.

^3^Center of Genetic & Prenatal Diagnosis, First Affiliated Hospital, Zhengzhou University, Zhengzhou, 450052, China.

^4^Department of General Surgery, Tangdu Hospital, Fourth Military Medical University, Xi'an, 710032, China.

^5^ Department of General Surgery, Huaihai Hospital, Xuzhou Medical University, Xuzhou, 221004, China

**Supplementary Figures**

**
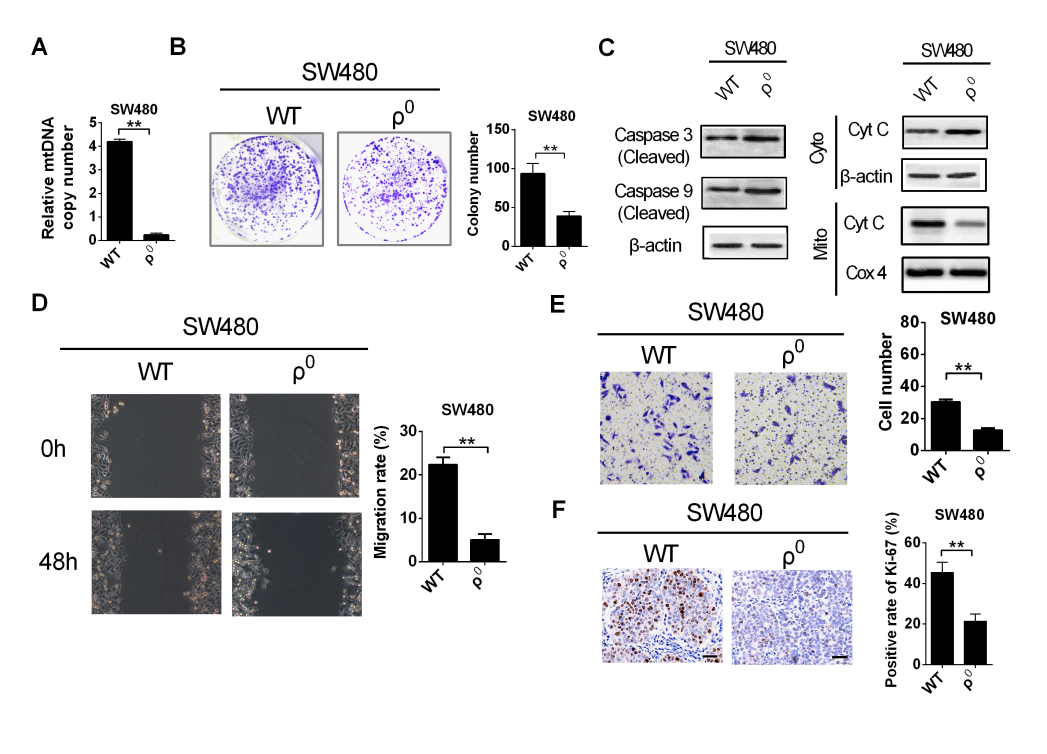
**

**Figure S1( Related to Figure 5). (A)** qRT–PCR analyses for mtDNA copy number was performed in SW480 cell and mtDNA depleted SW480 cell (ρ^0^ cell) line. **(B)** Colony formation assay in SW480 cells with different treatment as indicated. **(C)** Western blot analyses for protein levels of caspase3/9 and cytochrome C (Cyt C)in SW480 cells with treatment as indicated.SW480 cells and ρ^0^ cells were treated with CCCP (150 μM) for 4 hours before analysis. β-actin and Cox 4 were used as loading controls for cytoplasm and mitochondria, respectively. Cyto: cytoplasm, Mito: mitochondria. **(D)** Representative images of the wound healing assay in SW480 cells with treatment as indicated. **(E)** Transwell migration analysis for SW480 cells with treatment as indicated. **(F)** Representative immunohistochemical (IHC) staining images of Ki-67 in xenograft tumors developed from SW480 cells with treatment as indicated. Scale bar: 50 μm. ** *P* < 0.01.

**
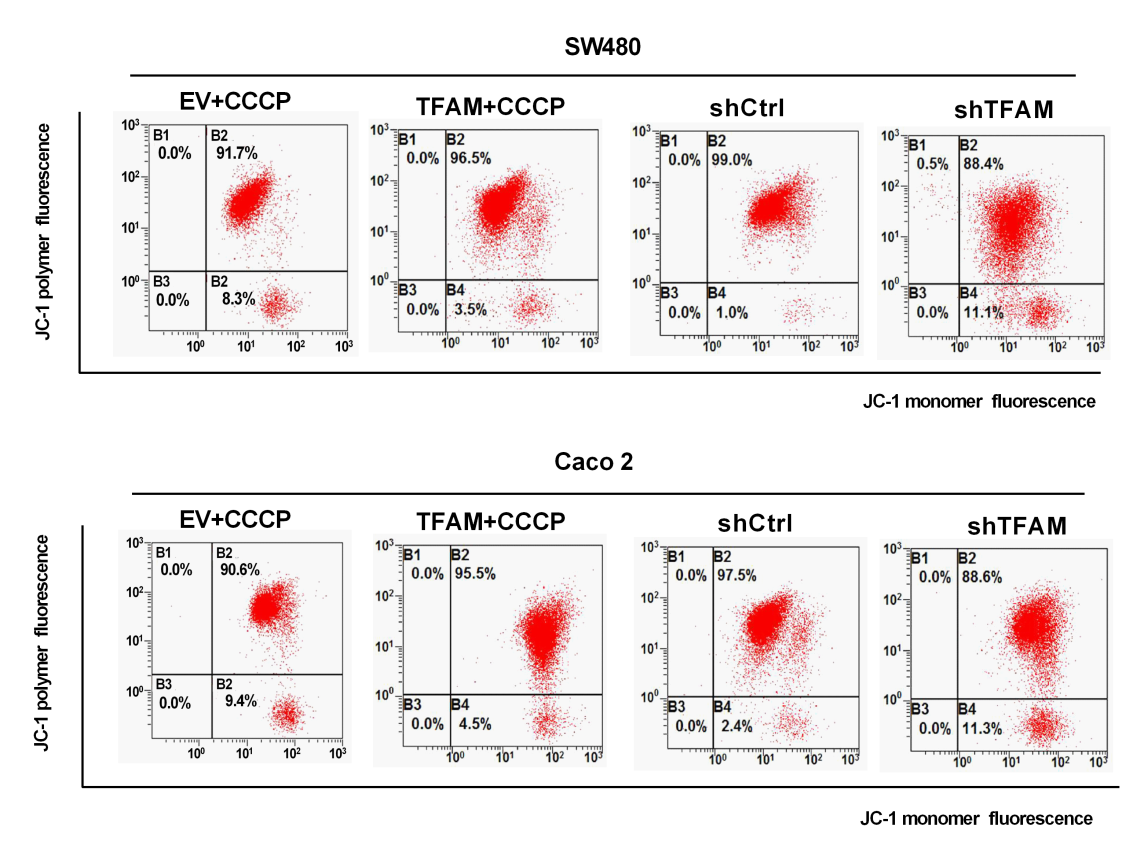
**

**Figure S2( Related to Figure 6)**. Depolarization of mitochondrial membrane potentials was analyzed by JC-1 staining in SW480 and Caco 2 cells with treatment as indicated. Cells stably transfected with EV or TFAM vector were also treated with CCCP (150 μM) for 4 hours before apoptosis analysis. TFAM, expression vector encoding TFAM; EV, empty vector; shTFAM, shRNA vector against TFAM; shCtrl, control shRNA vector.

**Supplementary table**

| **1. Primers used in q-PCR analysis** | | | |
| --- | --- | --- | --- |
| *ND1* | forward primer | CCCTAAAACCCGCCACATCT |  |
|  | reverse primer | GAGCGATGGTGAGAGCTAAGGT |  |
| *HGB* | forward primer | GTGCACCTGACTCCT GAGGAGA |  |
|  | reverse primer | CCTTGATACCAACCTGCCCAG |  |
|  | | |  |
| **2. Primers used in gene cloning** | | |  |
| *TFAM* | forward primer | GCGGATCCATGGCGTTTCTCCGAAGCATGTG |  |
|  | reverse primer | GCGAATTCttaacactcctcagcaccatattttcg |  |
|  |  |  |  |
| **3. siRNA** |  |  |  |
| *TFAM* siRNA | sense | GATCCGTTGTCCAAAGAAACCTGTTTCAAGAGAACAGGTTTCTTTGGACAACTTTTTTGGAAA | |
|  | antisense | AGCTTTTCCAAAAAAGTTGTCCAAAGAAACCTGTTCTCTTGAAACAGGTTTCTTTGGACAACG | |
| Control siRNA | sense | UUCUCCGAACGUGUCACGUTT | |
|  | antisense | ACGUGACACGUUCGGAGAATT | |
